# CD133 facilitates epithelial-mesenchymal transition through interaction with the ERK pathway in pancreatic cancer metastasis

**DOI:** 10.1186/1476-4598-13-15

**Published:** 2014-01-27

**Authors:** Qiang Ding, Yumi Miyazaki, Koichiro Tsukasa, Shyuichiro Matsubara, Makoto Yoshimitsu, Sonshin Takao

**Affiliations:** 1 Division of Cancer & Regenerative Medicine Kagoshima University Graduate School of Medical and Dental Sciences, 8-35-1, Sakuragaoka, Kagoshima 890-8520, Japan; 2 Department of Hematology and Immunology, Kagoshima University Graduate School of Medical and Dental Sciences, 8-35-1, Sakuragaoka, Kagoshima 890-8520, Japan

**Keywords:** Pancreatic cancer, CD133, Epithelial-mesenchymal transition (EMT), ERK1/2, N-cadherin, Cancer metastasis

## Abstract

**Background:**

Pancreatic cancer is a lethal disease due to the high incidence of metastasis at the time of detection. CD133 expression in clinical pancreatic cancer correlates with poor prognosis and metastasis. However, the molecular mechanism of CD133-regulated metastasis remains unclear. In recent years, epithelial-mesenchymal transition (EMT) has been linked to cancer invasion and metastasis. In the present study we investigated the role of CD133 in pancreatic cancer metastasis and its potential regulatory network.

**Methods:**

A highly migratory pancreatic cancer cell line, Capan1M9, was established previously. After shRNA was stable transducted to knock down CD133 in Capan1M9 cells, gene expression was profiled by DNA microarray. Orthotopic, splenic and intravenous transplantation mouse models were set up to examine the tumorigenesis and metastatic capabilities of these cells. In further experiments, real-time RT-PCR, Western blot and co-immunoprecipitate were conducted to evaluate the interactions of CD133, Slug, N-cadherin, ERK1/2 and SRC.

**Results:**

We found that CD133^+^ human pancreatic cancer cells were prone to generating metastatic nodules in in vivo models using immunodeficient mice. In contrast, CD133 knockdown suppressed cancer invasion and metastasis in vivo. Gene profiling analysis suggested that CD133 modulated mesenchymal characteristics including the expression of EMT-related genes, such as Slug and N-cadherin. These genes were down-regulated following CD133 knockdown. Moreover, CD133 expression could be modulated by the extracellular signal-regulated kinase (ERK)1/2 and SRC signaling pathways. The binding of CD133 to ERK1/2 and SRC acts as an indispensable mediator of N-cadherin expression.

**Conclusions:**

These results demonstrate that CD133 plays a critical role in facilitating the EMT regulatory loop, specifically by upregulating N-cadherin expression, leading to the invasion and metastasis of pancreatic cancer cells. Our study provides a novel insight into the function of CD133 in the EMT program and a better understanding of the mechanism underlying the involvement of CD133 in pancreatic cancer metastasis.

## Background

Pancreatic ductal adenocarcinoma (PDAC) is a lethal disease due to the high occurrence of metastasis at the time of detection [1]. The poor prognosis of patients with PDAC has been attributed to early vascular dissemination and metastasis to distant organs, particularly the liver, lungs, and peritoneum. Accumulating evidence has shown that the increased motility and invasiveness of cancer cells are associated with epithelial-mesenchymal transition (EMT) [2]. EMT is a cellular program that governs morphogenesis and is activated during embryogenesis. EMT transcription factors mediate the conversion of polarized immotile epithelial cells into mobile mesenchymal progenitor cells, which can facilitate cancer cell migration and invasion. Several groups have reported that EMT also contributes to the properties of cancer stem cells (CSCs) [3-5]. For example, Mani et al. demonstrated that immortalized human mammary epithelial cells undergoing EMT not only resulted in the acquisition of mesenchymal traits but also expressed stem cell markers and acquired the properties associated with mammary epithelial stem cells [5].

Dysregulation of the EMT program contributes to tumor initiation, invasion, and metastatic spread and an increase in tumor stemness. The primary molecular feature of carcinoma EMT is the upregulation of characteristic mesenchymal genes, including N-cadherin, fibronectin, snail1, and snail2 (Slug). EGF signaling pathways are also potent inducers of the EMT program [6], and EGF can activate several pathways through its receptor. The two major intracellular pathways activated by EGFR are the RAS-RAF-MEK-MAPK-ERK pathway and the PI3K-Akt pathway [7].

Oncogenic Kras is the initiating mutation in nearly all PDACs. The Ras-ERK pathway is involved in subsequent malignant proliferation, migration, and invasion. According to reports, most PDACs also have elevated SRC activity [8]. CD133 and SRC kinase have recently been shown to play roles in the regulation of tumor-initiating properties and the EMT program of head and neck carcinoma cells [9]. As SRC is a classical non-receptor tyrosine kinase with the potential to cause cell transformation, CD133 may play an important role in the regulation of SRC function by acting as a substrate for SRC family tyrosine kinases [9,10].

The transmembrane protein CD133 (also known as prominin-1 or AC133) is of particular interest and a subject of much debate. CD133 is the most commonly expressed CSC marker in several cancer types, including pancreatic cancer. It has been reported that CD133^+^ cells are the dominant cell population in primary non-small cell lung cancer and that these cells have greater tumorigenic potential in severe combined immunodeficient (SCID) mice and greater involvement in stemness, adhesion, and motility in comparison to their CD133^-^ counterparts [11]. However, the biological function of CD133 in the metastasis of solid carcinomas is still unknown.

We have previously reported a significant correlation between CD133 expression and clinicopathologic factors, histological type, lymphatic invasion, and lymph node metastasis in a cohort of pancreatic cancer patients who underwent curative surgery [12]. We were specifically able to select a subclone from a pancreatic cancer cell line that reflected aggressive migratory behavior and high CD133 expression. CD133 plays a regulatory role in the expression of Slug, which is one of the major EMT transcription factors [13]. These results strongly imply that CD133 participates in the regulatory network to facilitate EMT. The goal of the present study was to unravel the molecular mechanisms involved in the regulatory association between EMT and CD133 in the interaction network.

## Results and discussion

### CD133 contributes to hematogenous metastasis in vivo

**Figure 1:**
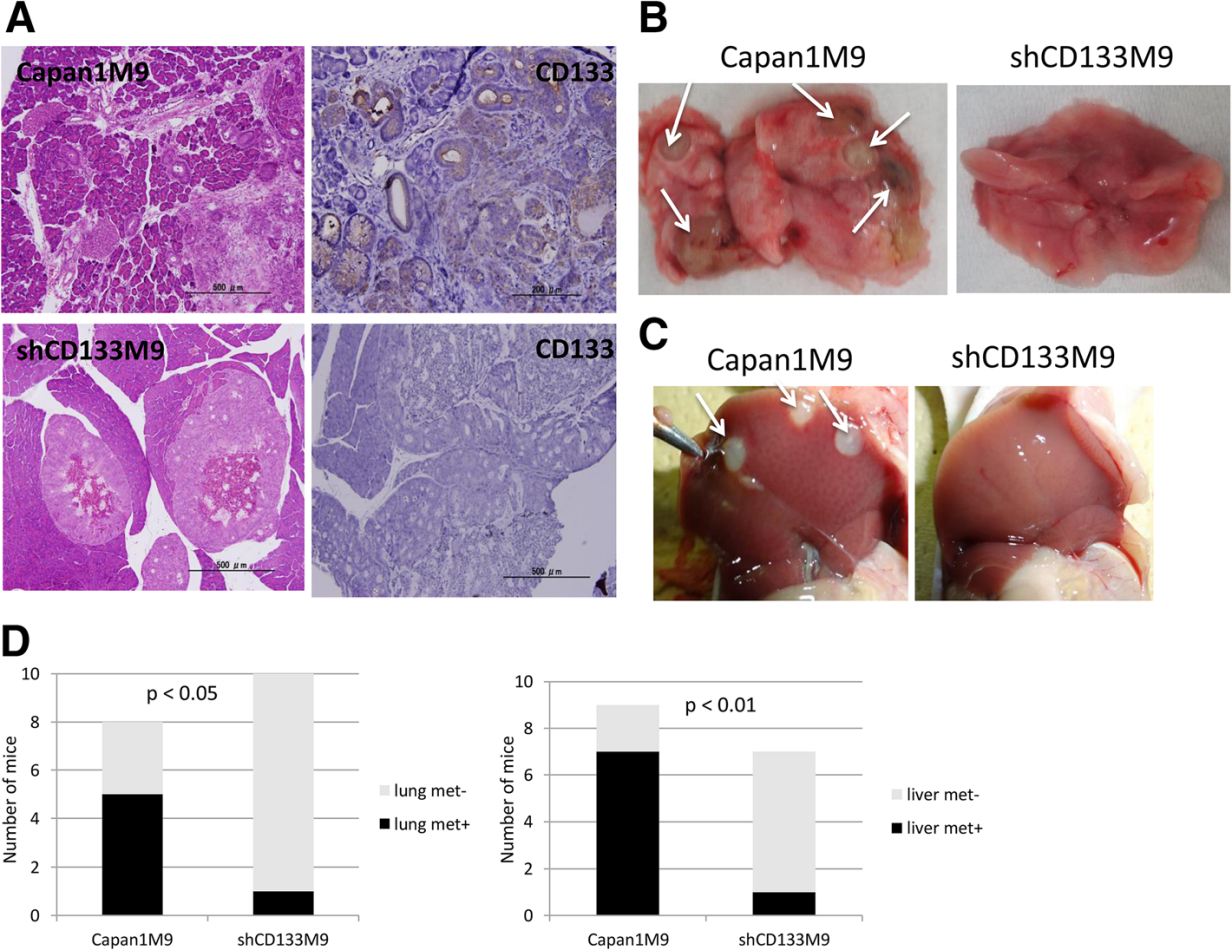
**CD133**^**+ **^**pancreatic cancer cells were prone to generating metastatic nodules in in vivo models using immunodeficient mice. (A)** Tumor morphologies (*left*) and CD133 immunohistological staining (*right*) of these tumors, which were observed in the pancreas at 4 weeks after the orthotopic implantation of Capan1M9 (CD133^high^) or shCD133M9 (CD133^KD^) cells. **(B)** Macro-metastatic nodules were observed in the lungs 16 weeks after the intravenous injection of Capan1M9 cells into the tail vein (*left*). The nodule was barely visible after the injection of shCD133M9 cells (*right*). **(C)** Macro-metastatic nodules observed in the liver at 4 weeks after the splenic implantation of Capan1M9 cells (*left*). In contrast, the metastatic nodule was barely visible after the implantation of shCD133M9 cells (*right*). **(D)** Statistical analysis of the number of macro-metastatic nodules generated in the lungs or liver after intravenous or splenic implantation showed that Capan1M9 cells were prone to generating metastases in comparison with shCD133M9 cells. Each mouse received 2 × 10^5^ cells of Capan1M9 or shCD133M9 in these experiments.

In our previous study, we reported that a highly migratory subclone, Capan1M9, derived from the human pancreatic cancer cell line, Capan-1, exhibited a high level of CD133 expression (Additional file 1: Figure S1). The migratory ability of these cells was suppressed by knockdown of CD133 expression with shRNA in vitro [13]. To investigate the different capabilities of CD133^high^ (Capan1M9) and CD133^knockdown (KD)^ (shCD133M9) cells in tumorigenesis and metastasis, we established an orthotopic pancreatic tumor model to examine local invasion and a hematogenous lung or liver metastasis model using BALB/c nude mice. More specifically, a dose of 2×10^5^ Capan1M9 or shCD133M9 cells was injected into nude mice. Following injection into the pancreas, spleen, or tail vein, the mice were sacrificed at 4, 4, or 16 weeks after cell implantation, respectively (Additional file 1: Figure S2). As determined by hematoxylin and eosin (HE) staining, the tumors generated by Capan1M9 and shCD133M9 cells in the pancreas with cell morphology analogous to that of PDAC (Figure 1A). Moreover, the tumors generated by Capan1M9 cells also exhibited CD133-positive staining and an invasive and aggressive growth pattern. Many cancer lesions were observed invading the surrounding normal tissue, showing an intensive interaction with the stromal tissues (Figure 1A, top right). In contrast, the tumors generated by shCD133M9 cells exhibited CD133-negative staining and an expansive growth pattern with a clear margin between the tumor and surrounding stromal tissues (Figure 1A, bottom right). Moreover, five (62.5%) of the eight mice intravenously injected with Capan1M9 cells developed metastatic nodules in the lungs in comparison to one (10%) of the ten mice injected with shCD133M9 cells (Figure 1B, Figure 1D and Additional file 1: Figure S3). In the liver, seven (78%) of nine mice with splenic implantation of Capan1M9 cells generated metastatic nodules compared to one (12.5%) of eight mice injected with shCD133M9 cells (Figure 1C and Figure 1D). However, there was no significant difference of subcutaneously tumor-take rates between CD133^high^ and CD133^KD^ cells and the growth rates between the tumors generated by CD133^high^ and CD133^KD^ cells (Additional file 1: Figure S4 and S5). Moreover there was no significant difference of the tumor volumes between the tumors generated by CD133^high^ and CD133^KD^ cells in orthotopic tumor model after four weeks (Additional file 1: Figure S6). Execution of metastasis is composed of multiple processes including cancer cell detachment from the primary tumor, local invasion to disseminate through surrounding blood vessels or lymphatic vessels, and attachment and proliferation at the metastatic site. In our study, it is thought that CD133 contributes to the cancer cell survival in circulation, extravasation, colonization in the distant organ and formation of macro metastasis, which are essential for the process of cancer metastasis. It is also notable that there was no significant difference of the tumorigenesis capability between Capan1M9 and shCD133M9 cells. These results indicated that CD133 plays a critical role in tumor invasiveness and metastasis, but little role in tumorigenesis.

### CD133 as a regulator of the mesenchymal phenotype

**Figure 2:**
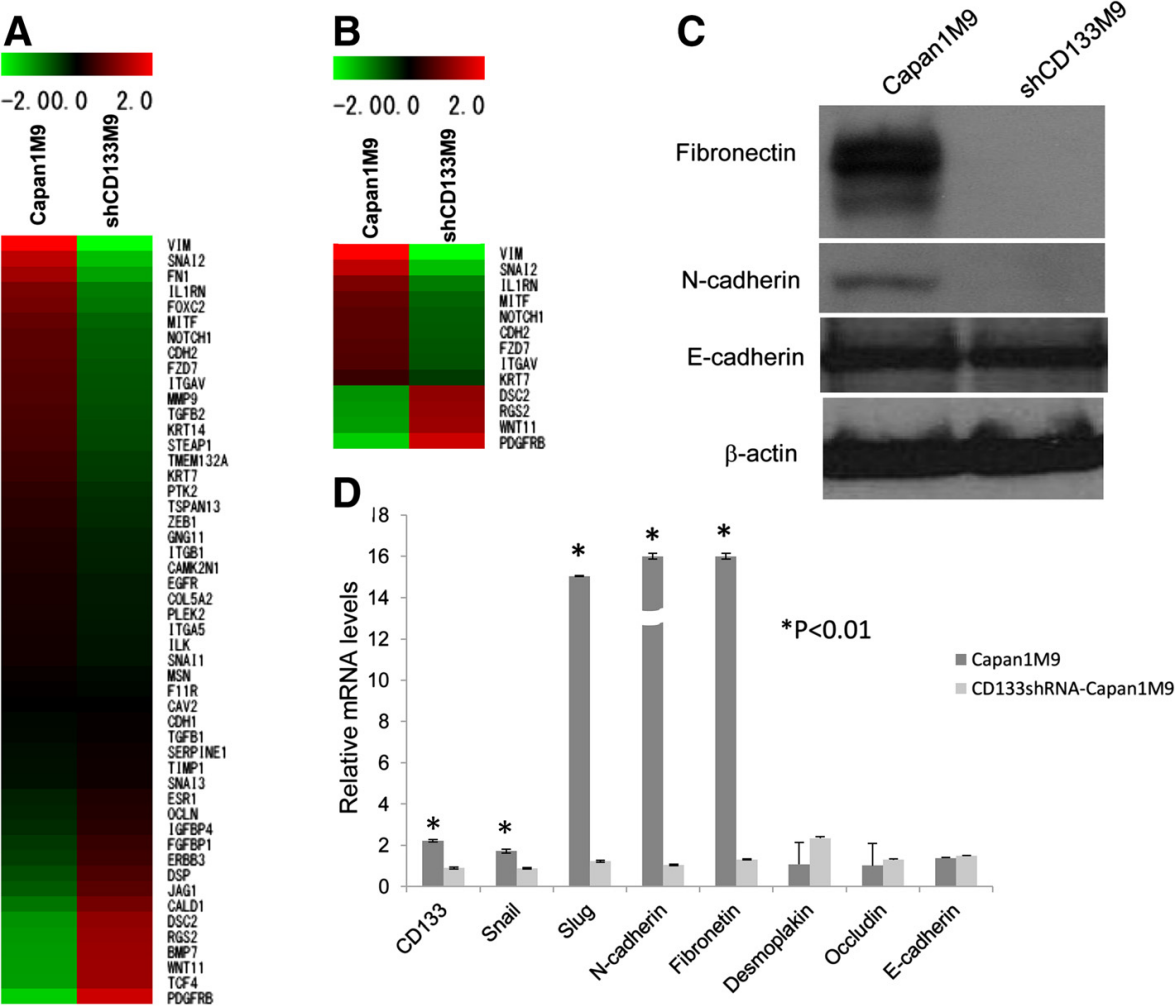
**CD133 acts as a regulator of the mesenchymal phenotype. (A)** A heatmap of the DNA microarray demonstrates the distinct molecular profiles of Capan1M9 and shCD133M9 cells. **(B)** A heatmap of EMT-related genes with a Z-score greater than 2 distinguished between Capan1M9 and shCD133M9 cells. **(C)** Validation of the levels of the EMT-related proteins fibronectin, N-cadherin, and E-cadherin in Capan1M9 and shCD133M9 cells by Western blot. **(D)** Validation of EMT-related genes in Capan1M9 with shCD133M9 cells by real-time RT-PCR.

To gain a genome-wide understanding of CD133-related gene expression in the highly migratory PDAC cells, we performed global gene-expression profiling in Capan1M9 and shCD133M9 cells. We used and improved an unbiased algorithm developed by Cheadle et al. [14] to assess whether a particular gene set was statistically over- or under-expressed in a given mRNA transcript expression profile. Briefly, we calculated Z-scores for each gene in a given expression profile, assuming that these expression values have a normal distribution to minimize the noise arising from different expression profiles obtained across diverse platforms. Microarray analyses indicated that the knockdown of CD133 led to a reduction in several molecular features of EMT. The profile of Capan1M9 (CD133^high^) or shCD133M9 cells (CD133^KD^) was clustered into two separate subgroups, as shown in Figure 2A and Additional file 2: Table S1. Slug, N-cadherin, and fibronectin, which are characteristic components of mesenchymal cells, were downregulated in the CD133^KD^ cells (Figure 2B and Additional file 2: Table S2). In contrast, E-cadherin, desmoplakin, and occludin, which are characteristic components of epithelial cells, showed no significant changes after CD133 knockdown. These findings were confirmed by real-time RT-PCR and Western blot analysis (Figure 2C and Figure 2D). Based on the above analysis and results, despite CD133 knockdown, shCD133M9 cells could still generate tumors in the orthotopic mouse models, suggesting that the epithelial phenotype plays the dominant role in tumorigenesis. This suggests that only cells possessing mesenchymal traits can infiltrate into the stroma, invade, and disseminate to distant organs. Gene profiling analysis suggested that CD133 might be a regulator of mesenchymal markers. Evidence shows that during EMT, the activity of adherent junctions is highly modified, primarily because E-cadherin is replaced or overruled by N-cadherin in a process called “cadherin switching” [15]. Our data implied that N-cadherin has the dominant effect in these cell-cell interactions, even in the presence of E-cadherin. Over-expressed N-cadherin may disrupt the intercellular adhesion complexes and result in the epithelial cell losing the apical-basal polarity. These changes enable the epithelial cells to assume a mesenchymal phenotype and to enhance the motility of tumor. It is so-called the initial EMT program. CD133 is a transmembrane protein, which location is well suited for a cell protrusion to participate the signaling pathway regulating the EMT program, particularly the “cadherin switch”.

### CD133 and Slug regulate the N-cadherin expression required for migration and invasion

**Figure 3:**
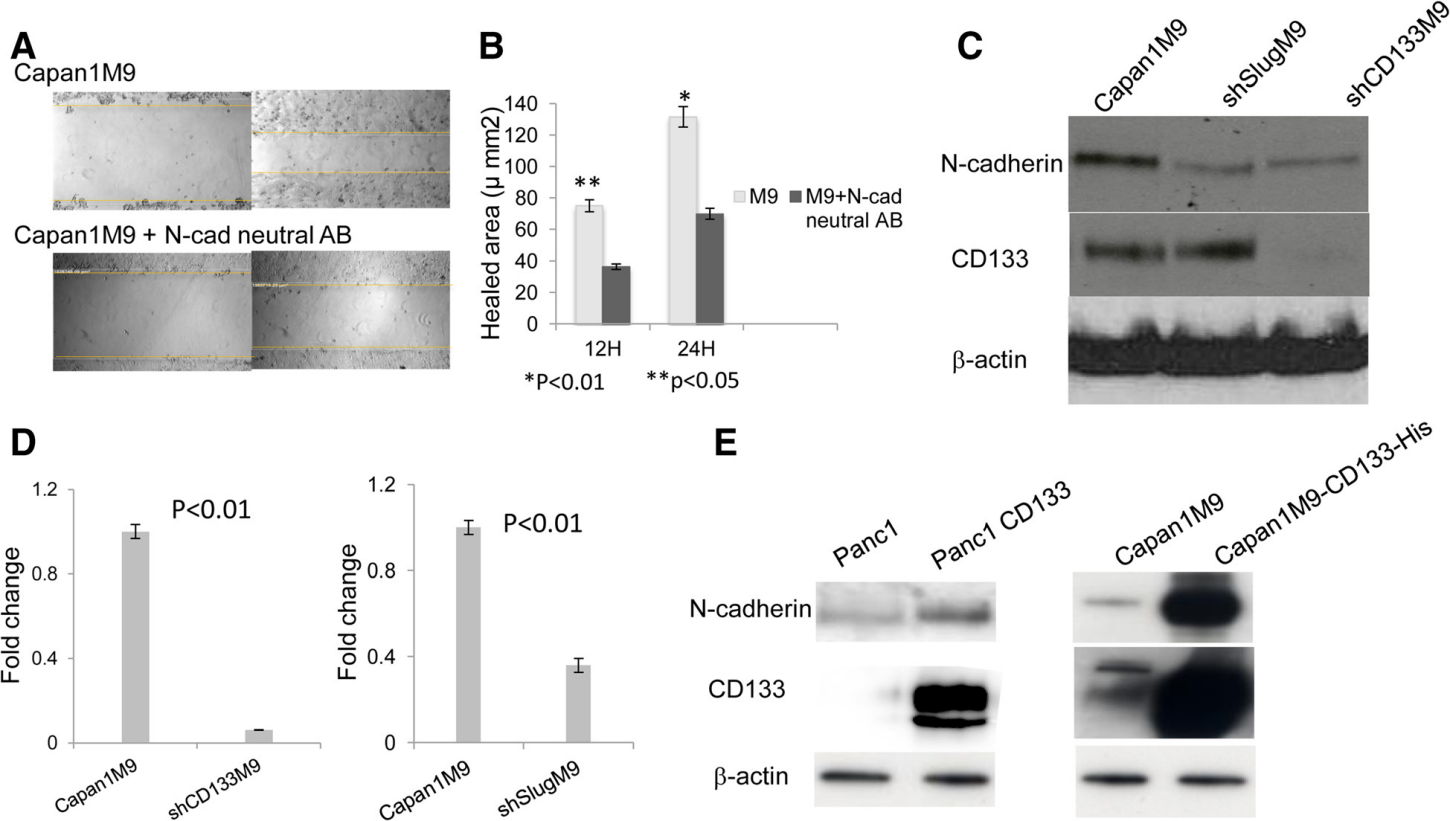
**CD133 and Slug regulate N-cadherin expression, which is required for migration and invasion. (A)** A wound healing assay showed that the healing speed decreased after N-cadherin neutralization using an antibody. **(B)** Statistical analysis of the mean healed area demonstrated significant differences between with and without N-cadherin antibody neutralization at 12 h (p < 0.05) and 24 h (p < 0.01). **(C)** N-cadherin mRNA expression decreased by greater than 95% and 60% after CD133 and Slug knockdown, respectively, validated by real-time RT-PCR. **(D)** N-cadherin protein expression decreased after CD133 or Slug knockdown, as shown by Western blotting. **(E)** N-cadherin protein expression increased after CD133 was overexpressed in Capan1M9 cells and Panc-1 cells, as shown by Western blot analysis.

Reports have demonstrated that N-cadherin can promote motility, invasion, and metastasis in breast cancer, even in the presence of E-cadherin [15]. To investigate the role of N-cadherin in PDAC cell migration and invasion, Capan1M9 was treated with an N-cadherin-neutralizing antibody, and a wound healing assay was performed. After the N-cadherin-neutralizing antibody was introduced, the wound healing speed was significantly slower than that without antibody neutralization. The healing area in the antibody-treated group was half of that without neutralization (Figure 3A and Figure 3B). To investigate whether CD133 and Slug could regulate N-cadherin expression in pancreatic cancer during migration and invasion, shRNA was transduced into Capan1M9 cells to knockdown Slug or CD133. Real-time RT-PCR was used to validate the efficiency after transduction. The expression levels of Slug and CD133 were reduced by 70% and 60%, respectively (Additional file 1: Figure S7). The mRNA and protein expression levels of N-cadherin were determined by real-time RT-PCR and Western blot analysis, respectively, after shRNA transduction of CD133 or Slug. N-cadherin mRNA expression was decreased by greater than 95% and 60% after CD133 and Slug knockdown, respectively (Figure 3D). N-cadherin protein expression was also significantly decreased after CD133 or Slug knockdown (Figure 3C). In addition, the Slug expression level was reduced after CD133 knockdown, whereas the CD133 expression level did not change after Slug knockdown (Figure 3C).

To further confirm that CD133 plays roles in regulating N-cadherin and in PDAC migration and invasion, we overexpressed CD133 by transducing His-CD133 into Capan1M9 cells using lentiviral vectors. CD133 expression was upregulated following transduction as well as the N-cadherin expression level (Figure 3E). To exclude cell line-specific effects, we also overexpressed CD133 in Panc-1 cells with only 1.3% of CD133 expression. N-cadherin upregulation was detected in the His-CD133-transduced Panc-1 cells, consistent with the results obtained using Capan1M9 cells (Figure 3E).

These data indicate that the inhibition of N-cadherin alone could suppress the migratory ability of pancreatic cancer. According to studies in human prostate carcinoma, loss of E-cadherin expression and overexpression of N-cadherin, which indicate the presence of EMT, are independently correlated with a high Gleason score and systemic and metastatic recurrence after surgery. This finding links EMT to more aggressive clinical behavior [16]. In breast cancer, a similar link has been established between EMT markers in primary and disseminated bone marrow tumor cells and aggressive clinical behavior [17]. Furthermore, in liver cancer cell lines, CD133^+^ cells showed upregulated N-cadherin expression and downregulated E-cadherin expression compared with CD133-negative cells, which suggest that EMT more commonly occurs in CD133-positive cells than in CD133-negative cells [18]. The present study indicates that CD133 plays an important role in N-cadherin expression, and regulates Slug expression. Furthermore, Slug could regulate N-cadherin expression. However, there might be other molecules involved with CD133 in the regulation of the expression of Slug, which is a transcription factor. Further study is needed to clarify this regulation loop. It infers that CD133 may be a critical mediator facilitating EMT, primarily through Slug and N-cadherin regulation.

### The MAPK/ERK and SRC pathways underlie CD133 expression

**Figure 4:**
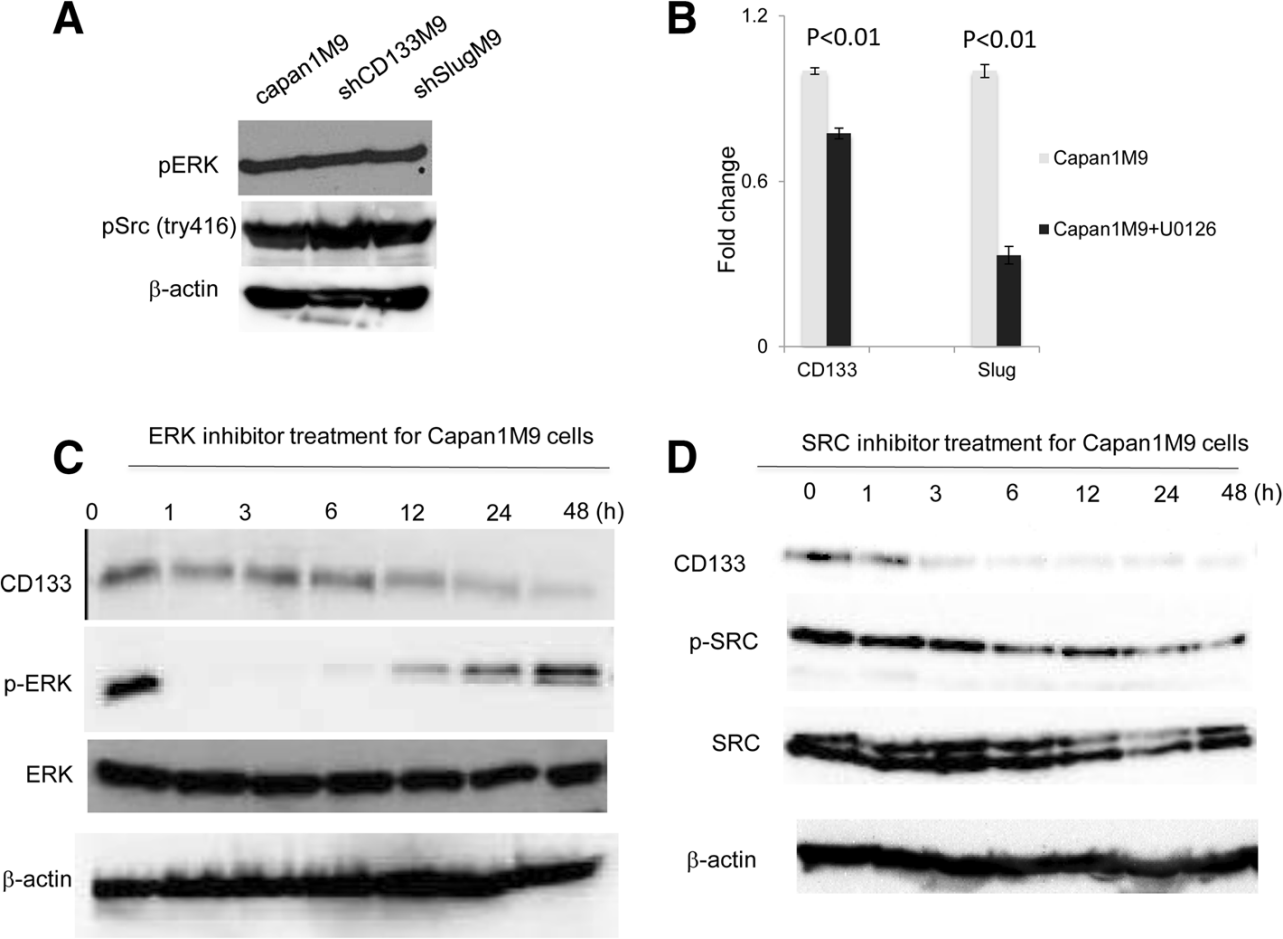
**The MAPK/ERK/SRC pathway underlies CD133 expression. (A)** No alteration of the protein expression levels of pERK and pSRC was detected inCapan1M9, shCD133M9 or shSlugM9 cells. **(B)** CD133 and Slug expression decreased after ERK1/2 inhibitor (10 μg/mL) treatment; results were validated by real-time RT-PCR. **(C)** CD133 protein expression decreased drastically after ERK1/2 inhibitor (10 μg/mL) treatment, as shown by Western blot analysis. **(D)** CD133 protein expression decreased dynamically after SRC inhibitor (10 μg/mL) treatment, as shown by Western blot analysis.

The MAPK/ERK pathway is a chain of proteins within cells, and it communicates a signal from a cell surface receptor to the DNA in the nucleus. The signal starts when a signaling molecule binds to the cell surface receptor and ends when the DNA in the nucleus expresses a protein that induces a certain change in the cell, such as cell division. The pathway includes many proteins, including MAPK (originally called ERK). Components of the MAPK/ERK pathway were initially discovered in many solid cancers, including PDAC [19,20]. ERK1/2 is the primary mediator among these components and is stimulated by EGF. It has also been shown that CD133 can be phosphorylated by SRC to activate its function [9]. To unravel the CD133-related signaling pathway in EMT regulation, we examined the effects of the classical EMT-inducing factors TGF and EGF on Slug and N-cadherin expression. Capan1M9 cells were exposed to TGF and the ERK1/2 inhibitors, SB431542 and U1026, and the expression of Slug and N-cadherin was examined. Compared with TGF inhibitor treatment, the ERK1/2 inhibitor showed a more powerful potential to decrease the expression of Slug and N-cadherin (Additional file 1: Figure S8). These results suggested that ERK could be the dominant factor triggering EMT in Capan1M9 cells. To investigate the relationship between ERK, SRC, and CD133, we examined the expression levels of phosphorylated ERK (p-ERK) and phosphorylated SRC (p-SRC) before and after CD133 knockdown. Western blot analysis detected no alteration after CD133 or Slug knockdown (Figure 4A). We then treated Capan1M9 cells with U0126 or a SRC inhibitor, and the mRNA and protein expression levels were examined. The mRNA levels of CD133 and Slug were reduced after the cells were treated with ERK inhibitor (U0126) for 48 h (Figure 4B). Additionally, CD133 protein expression was dynamically decreased in a time-dependent manner when cells were treated with ERK or SRC inhibitor (Figure 4C and Figure 4D).

The expression levels of p-ERK and p-SRC did not change significantly after CD133 or Slug knockdown, which suggested that the ERK or SRC pathway might be located upstream of the CD133 and Slug regulation loop. According to one report, CD133 expressed endogenously or exogenously in medulloblastoma cells is phosphorylated by SRC and Fyn, two members of the SRC-family tyrosine kinases [9]. In our study, CD133 expression in Capan1M9 cells was downregulated by a SRC inhibitor. Kemper et al. defined that a relationship between the hyper-activation of the Ras-Raf-MEK-ERK pathway can regulate CD133 expression, and mutations of either gene have been associated with a poor prognosis [21]. Our study is consistent with these reports and reveals the associations between ERK, SRC, and CD133.

### The ERK/SRC/CD133 axis as an indispensable complex regulating N-cadherin

**Figure 5:**
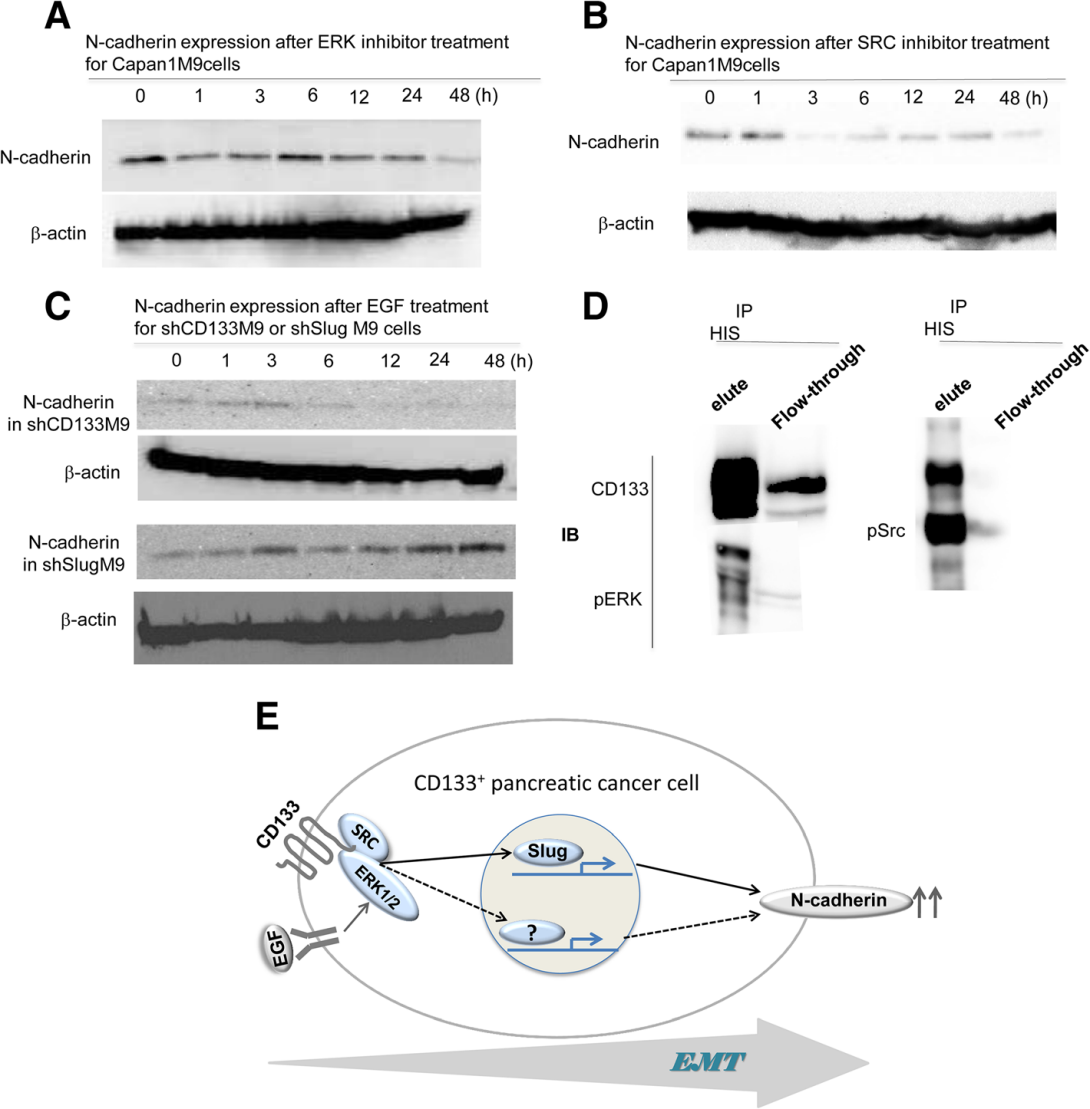
**ERK/SRC/CD133 is an indispensable complex for the regulation of N**-**cadherin. (A)** N-cadherin expression decreased dynamically after ERK1/2 inhibitor (10 μg/mL) treatment in Capan1M9 cells, as shown by Western blot analysis. **(B)** N-cadherin expression decreased dynamically after SRC inhibitor (10 μg/mL) treatment in Capan1M9 cells, as shown by Western blot analysis. **(C)** N-cadherin expression did not increase following EGF (10 μg/mL) treatment in shCD133M9 cells, although it did increase in shSlugM9 cells following EGF (10 μg/mL) treatment, as shown by Western blot analysis. **(D)** After transducing His-CD133 into Panc-1 and Capan1M9 cells, anti-His immunoprecipitates (IPs) were probed with anti-ERK1/2, anti-SRC, and anti-CD133 antibodies. IB, immunoblotting. **(E)** Hypothetical scheme of the CD133/ERK/N-cadherin regulation loop in pancreatic cancer metastasis. CD133 expression was upregulated, and CD133 combined with ERK and SRC to form a regulation complex that was indispensable for EGF activation of the ERK pathway and subsequent N-cadherin expression. CD133 acts as a mesenchymal mediator to facilitate the EMT program.

We found that CD133 could regulate Slug to facilitate EMT and regulate N-cadherin expression and that ERK and SRC could regulate CD133 expression. These findings raised the question of whether a network exists between ERK, SRC, and CD133 that is associated with N-cadherin regulation. We first examined N-cadherin expression after U0126 or SRC inhibitor treatment. A time-dependent decrease in N-cadherin expression was observed after these treatments in Capan1M9 cells (Figure 5A and Figure 5B). Further, we examined EGF stimulation of N-cadherin expression in shCD133M9 and shSlugM9 cells. The level of N-cadherin expression after EGF treatment did not increase in CD133^KD^ cells; on the contrary, it was increased in shSlugM9 cells (Figure 5C). A co-immunoprecipitation experiment was performed to unravel the relationship between CD133, ERK, and SRC. CD133-His was transduced into Capan1M9 cells, and we used an anti-His antibody to immunoprecipitate the cell lysate. CD133, ERK, and SRC were examined by immunoblotting. p-ERK and p-SRC (tyr416) were detected in the eluate from the immunoprecipitated His (Figure 5D). ERK1/2 is the primary mediator among the MAPK/MEK pathway components. Our observation revealed that CD133 is bound to ERK and SRC.

The preferential localization of CD133 to plasma membrane protrusions [22] and its ability to specifically interact with plasma-membrane cholesterol in cholesterol-based lipid rafts suggest the involvement of CD133 in the regulation of plasma membrane topology. To establish and maintain membrane protrusions, CD133 may be involved in cell polarity and migration [22] via cell-cell and cell-extracellular matrix interactions [23]. According to many reports, EGF is the dominant factor that increases N-cadherin levels [21,24]. Upregulation of N-cadherin was observed in shSlugM9 cells following EGF treatment, but knockdown of CD133 abolished the ability of EGF to induce N-cadherin expression (Figure 5C). These data indicate that CD133 is an indispensable mediator of N-cadherin expression (Figure 5E).

## Conclusions

Wei and colleagues have recently reported that the CD133/PI3K/Akt signaling axis regulates glioma stem cell behavior, self-renewal, and tumorigenesis, as SRC binds to the cytoplasmic tail of CD133 and consequently activates the PI3K/Akt pathway [25]. CD133 expression could be upregulated by activated ERK and SRC, and form a CD133/ERK/SRC complex, which is necessary for EGF to activate the downstream ERK pathway and induce N-cadherin expression and facilitate the EMT program (Figure 5E). The presence of the CD133/ERK/SRC signaling axis indicates that CD133 acts as a characteristic mesenchymal regulator and is a “functional” marker of migration, invasion, and metastasis in pancreatic cancer. Therefore, CD133 plays a critical role in survival of pancreatic cancer cells in the circulation system, extravasation, and colonization in the metastatic sites. Our study provides novel insight into the interaction between CSCs and the EMT program and a better understanding of the mechanism underlying the involvement of CD133 in cancer metastasis. Furthermore, our study may facilitate the discovery of a novel targeted therapy and diagnostic tool for PDAC.

## Methods

### Cells and reagents

The human pancreatic cancer cell lines Capan-1 and Panc-1 were purchased from the American Type Culture Collection (ATCC, VA, USA). A highly migratory subclone cell line, Capan1M9, was established from the Capan-1 as previous reported [13]. EGF, an ERK1/2 inhibitor (U0126), a SRC inhibitor-1, and a TGF inhibitor (SB431542) were used at a working concentration of 10 μg/mL; all were purchased from Sigma-Aldrich Co. (St. Louis, MO, USA).

### Animal studies

Male BALB/c nude mice aged 8 to 10 weeks were purchased from Crea Japan Co. Ltd. (Tokyo, Japan).

1. Orthotopic and metastatic mice models:

To create an orthotopic tumor model in the pancreas and a liver or pulmonary metastasis model, tumor cells were implanted into the pancreas, spleen, or lateral tail vein via a 27-gauge needle under anesthesia, which was induced by the inhalation of 1-chloro-2,2,2-trifluoroethyl difluoromethyl ether (Isoflurane). The mice were immobilized in a restraining device. Tumor cell implantations were performed using freshly prepared suspensions at a concentration of 2 × 10^6^ tumor cells/mL, and 2 × 10^5^ tumor cells were implanted into each nude mouse. The design of the mouse experiment is shown in Additional file 1: Figure S2. After implantation, mouse body weight was measured once a week. The nude mice were sacrificed 4 weeks after pancreas or spleen implantation or 16 weeks after tail vein injection. Organs, including the liver, pancreas, and lungs, were fixed in formalin for standard paraffin embedding. The resultant 3-μm paraffin sections were fixed and stained with hematoxylin-eosin (HE).

2. Subcutaneously xenograft mice model

1×10^2^ or 1×10^3^ tumors cells were inoculated subcutaneously at the flanks of mice (Additional file 1: Figure S4). Tumor size was monitored twice a week after the visible tumor nodules appeared.

All animal experiments were approved by the Committee on the Use of Live Animals for Teaching and Research and conducted in accordance with the Animal Care and Use Committee guidelines of Kagoshima University.

### Wound healing assay

A CytoSelect™ 24-well wound healing assay (Cell Biolabs, CA, USA) was used as a migration assay. A wound field was generated according to the product manual. A cell suspension was added to the well, with the insert in place, and then incubated for 24–48 h. Next, the cells were cultured until a monolayer formed, and the insert was removed to generate a “wound field”. The cells were then monitored under a microscope to examine migration into the wound field until the wound closed. The wound healing area was calculated using the software AxioVisionRel (Zeiss, Germany).

### Immunohistochemical staining

Five consecutive 3-μm histological sections of the mouse tissues were stained with either HE, mouse monoclonal GFP antibody (Novus Biologicals, Littleton, USA), monoclonal mouse anti-human pan-cytokeratin (CK) (Dako, Carpinteria, CA, USA) or CD133/1 (AC133) (MiltenyiBiotec, Bergisch Gladbach, Germany). Briefly, after the 3-μm sections were deparaffinized and endogenous peroxidases were blocked, the sections were incubated at 4°C overnight with each antibody. The sections were incubated for 30 min with biotinylated anti-mouse IgG in PBS at room temperature. After being washed, the sections were incubated for 30 min with avidin and a biotinylated horseradish peroxidase complex, and immune complexes were visualized by incubating the sections with 3,3’-diaminobenzidine tetrahydrochloride (DAB) or PermaRed/AP (Diagnostic BioSystems, Pleasanton, CA, USA). Images were captured with an Olympus microscope. PBS was substituted for the primary antibody in the negative-control group.

### Cell lysates and immunoblotting

Cells were lysed on ice in lysis buffer. The lysates were then boiled for 5 min, clarified by centrifugation at 15,000 × *g* for 15 min, and separated by SDS-PAGE. The proteins were then transferred onto nitrocellulose membranes, which were incubated with a 1:100–200 dilution of human polyclonal or monoclonal antibodies raised against the following: E-cadherin, N-cadherin, pERK, ERK (Santa Cruz, CA, USA), fibronectin(R&D, MN, USA), CD133 (MiltenyiBiotec, Germany), and pSRC (CST, MA, USA). Next, a 1:200–1000 dilution of peroxidase-conjugated anti-goat IgG, anti-rabbit IgG (Santa Cruz, CA, USA), or anti-mouse IgG (Jackson ImmunoResearch, PA, USA) antibody was applied for the secondary reaction. As an internal control for protein loading, β-actin was detected using a specific antibody (Sigma, MO, USA). Immune complexes were visualized using the ECL Western blotting detection system (Amersham, UK).

### *shRNA and His*-*tag transfection*

The Capan1M9-GFP-shRNA CD133 cell line was established as previously described [13]. The Capan1M9-GFP-shSlug cell line was established by lentiviral transduction. pLVTHM is a second-generation lentiviral vector that engineers shRNA under an H1 promoter (Addgene, MA, USA) and co-expresses enGFP under the elongation factor 1a promoter. Slug shRNA sense (5’-cgcgtcccccagacccattctgatgtaaagttcaagagactttacatcagaatgggtctgtttttggaaat-3’) and Slug shRNA antisense (5’-cgatttccaaaaacagacccattctgatgtaaagtctcttgaactttacatcagaatgggtctggggga-3’) oligonucleotides were annealed to each other and ligated into the pLVTHM vector at the ClaI and MluI sites, which yielded the pLVTHM-Slug shRNA transfer vector. C-terminally His-tagged CD133 expressing the lentiviral vector was constructed by replacing the pDY.LNGFRTmpk lentiviral vector (2) with CD133-His cDNA. Briefly, CD133 cDNA was amplified with primer 1 (5’-GGTACCGCGGGCGCGCCATGGCCCTCGTACTCGGCTC-3’) and primer 2 (5’-ATTGAAGCTTGGATCCTCAGTGATGGTGATGGTGATGATGTTGTGATGGGCTTGTCA-3’) to add a His tag. The resultant amplicon was ligated into the pDY.LNGFRTmpk lentiviral vector by replacing TmpkcDNA with CD133-His cDNA using an In-Fusion HD Cloning Kit (Clontech, Mountain View, CA), generating the pDY.LNGFR CD133-His lentiviral vector. Next, 293 T cells were co-transfected with 4 μg of transfer plasmid, 3 μg of psPAX2 packaging plasmid, and 1 μg of pMD2. G envelope plasmid using FuGene 6 transfection reagent (Roche, CA, USA). Twenty-four hours after transfection, the medium was replaced with fresh DMEM with10% FBS. Forty-eight hours after transfection, the viral supernatant was harvested and filtered through a 0.45-μm filter. Capan1M9 cells were then transduced with filtered viral supernatant containing 8 μg/mL protamine sulfate for 72 h after transfection. Flow cytometry analysis of enGFP expression was performed with a FACS can (BD Biosciences, CA, USA). enGFP-positive cell fractions were then sorted with a FACSAria (BD Biosciences, CA, USA). The purity of the fractions routinely exceeded 95%.

### *Co*-*immunoprecipitation and Western blotting*

Cells were washed three times with cold PBS, lysed using NP40 cell lysis buffer (Invitrogen, CA, USA), and clarified by centrifugation at 13,000 × *g* for 10 min at 4°C. A Dynabeads Protein A Immunoprecipitation Kit was used for immunoprecipitation according to the product’s protocol. Briefly, the Dynabeads were bound to anti-His antibody, incubated with rotation for 90 min at 4°C to obtain a Dynabead-Ab complex, mixed with cell lysate, and incubated overnight with rotation at 4°C. The Dynabead-Ab-Ag complex was then washed, and elution buffer was added to harvest the bound protein, which was separated by SDS-PAGE. The proteins were then transferred onto nitrocellulose membranes, which were incubated with a 1:100–200 dilution of human monoclonal antibodies against the following: CD133, pERK, and pSRC. Immune complexes were visualized using the ECL Western blotting detection system (Amersham, UK).

### *Quantitative real*-*time RT*-*PCR* (*ABI*)

Total RNA (tRNA) was extracted using an RNeasy extraction kit (Qiagen, Germany). Primers and probes were obtained from Applied Biosystems™ (Life Technologies, CA, USA) as Assay-on-Demand Gene Expression Products. Real-time RT-PCR was performed following the supplier’s directions. The PCR mixture (20 μl) contained 10 μl of 2x TaqMan Universal PCR Master Mix, 1 μl of 20x working stock of the gene expression assay mix, and 20 μg of tRNA. Real-time RT-PCR was performed using a StepOne Real-Time PCR System (Applied Biosystems, CA, USA). The reaction was performed in triplicate for each sample. The fluorescence of the PCR products was detected by the same apparatus. The number of cycles for the amplification plot to reach the threshold limit (Ct value) was used for quantification. Glyceraldehyde-3-phosphate dehydrogenase (GAPDH) was used as endogenous control.

### DNA microarray

tRNA was extracted using an RNeasy extraction kit (Qiagen, Germany). The cDNA was amplified, labeled, and hybridized to a 44 K Agilent 60-mer oligo microarray according to the manufacturer’s instructions. All hybridized microarray slides were then scanned by an Agilent scanner. Relative hybridization intensities and background hybridization values were calculated using Agilent Feature Extraction Software.

### Statistics

A statistical analysis was performed with the statistical package StatView (Version 5.0, SAS Institute, Inc.) and Excel (Microsoft, Washington). Data were compared using Student’s t test, one-way analysis of variance, the Mann-Whitney U test, and/or the Kruskal-Wallis test. All data are presented as the mean ± standard deviation. Differences between means were considered to be statistically significant at p < 0.05.

## Competing interest

The authors declare that they have no competing interest.

## Authors’ contributions

ST and QD designed research. QD, MY, YM, MH, SM, KT, and ST performed research. ST contributed new reagents or analytic tools. QD and ST analyzed data; and ST and QD wrote the paper. All authors read and approved the final manuscript.

## Supplementary Material

Additional file 1: Figure S1 Examination of CD133 expression before and after shRNA-CD133 transducted into Capan1M9 cells by flow cytometer. **Figure S2**. In vivo experimental schedules of this study using nude mice. We used Capan1M9-GFP or shCD133M9-GFP cells for these experimental models. We created the lung and liver metastasis models using tail vein or spleen implantation, respectively. In the lung metastasis model, nude mice were sacrificed at every 4 weeks. +: Macroscopically detectable tumor lesion in the organs, -: Undetectable tumor lesion in the organs. **Figure S3**. The lung metastasis model created by the tail vein injection of Capan1M9-GFP or shCD133M9-GFP cells into nude mice. GFP-positive spots (*arrow*) indicate micrometastatic lesions in the section of the lung 12 weeks after the tail vein injection of 1 × 10^5^ Capan1M9-GFP cells. Immunohistochemical staining for CK and CD133 was performed to identify micrometastatic lesions. **Figure S4**. Comparison of tumor-take rates between CD133^high^ and CD133^KD^ cells by subcutaneous xenograft tumor model. **Figure S5**. Comparison of tumor growth curves in nude mice among the tumors generated by CD133^high^ and CD133^KD^ cells by subcutaneous xenograft tumor model. **Figure S6**. Comparison of four week’s tumor volumes between tumors generated by CD133^high^ and CD133^KD^ cells in pancreatic orthotopic model. **Figure S7**. Transduction of shRNACD133 or shRNASlug into Capan1M9 cells. mRNA expression levels of CD133 and Slug were examined after shRNA transduction of CD133 or Slug into Capan1M9 cells (*left*). Fluorescent observation of GFP after shRNA transduction into Capan1M9 cells (*right*). **Figure S8**. Validation of Slug expression after ERK or TGF inhibitor treatment. Slug mRNA expression was examined after ERK or TGF inhibitor (U0126 or SB431542) treatment. Click here for file

Additional file 2: Table S1 DNA microarray analysis of epithelial-mesenchymal transition (EMT)-related genes. The data represent a comparison of EMT-related genes expressions between Capan1M9 and shCD133M9 cells by DNA microarray. **Table S2**. Analysis of EMT-related genes with the following Z scores: Z score ≥ 2 and ratio ≥ 1.5 or Z score ≤ -2 and ratio ≤ 0.66. Click here for file
