# Supplementary material for: CD133 facilitates epithelial-mesenchymal transition through interaction with the ERK pathway in pancreatic cancer metastasis
Source: Mol Cancer. 2014 Jan 27;13:15. doi: 10.1186/1476-4598-13-15 (PMC3931313; doi:10.1186/1476-4598-13-15)

Figure S1: Examination of CD133 expression before and after shRNA-CD133 transducted into Capan1M9 cells.

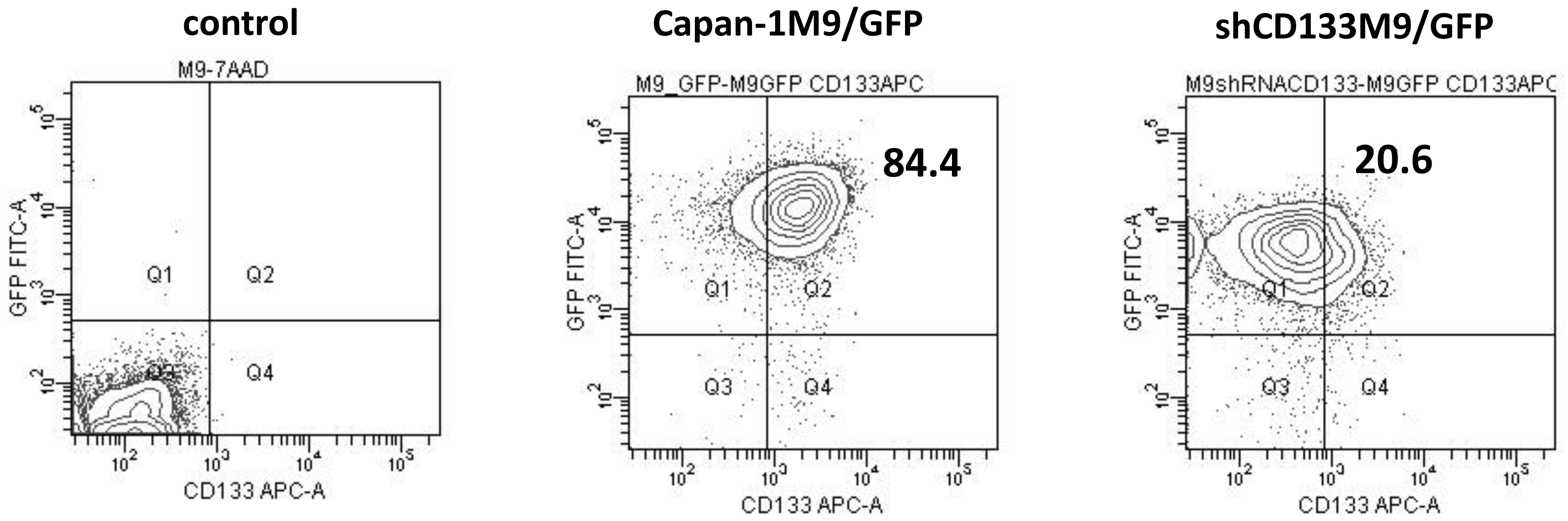

	CD133/1-APC	CD133/1-PE	GFP
Capan-1M9	89.0	72.6	0
Capan-1M9/GFP	84.4	72.6	99.0
shCD133M9/GFP	20.6	-	97.8

Figure S2: In vivo experimental schedule for analysis of invasion and metastasis by the metastatic or orthotopic models.

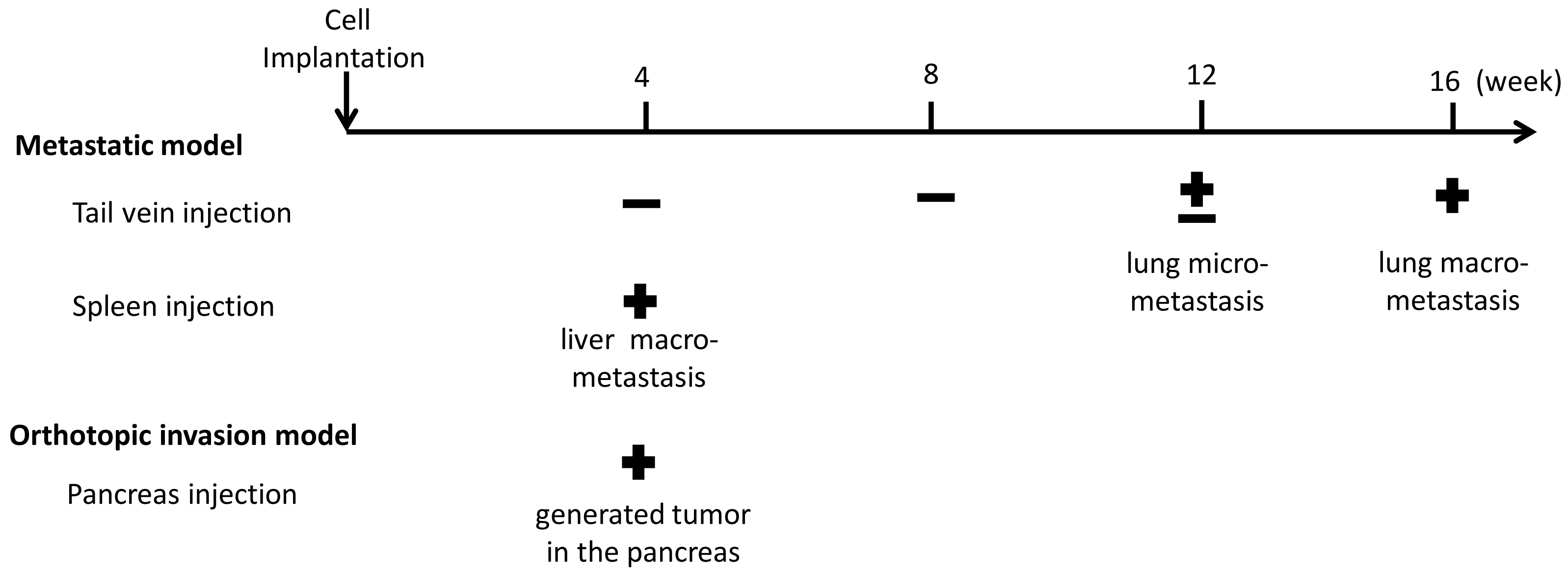

Figure S3: The lung metastasis model created by the tail vein injection of Capan1M9-GFP or shCD133M9-GFP cells into nude mice.

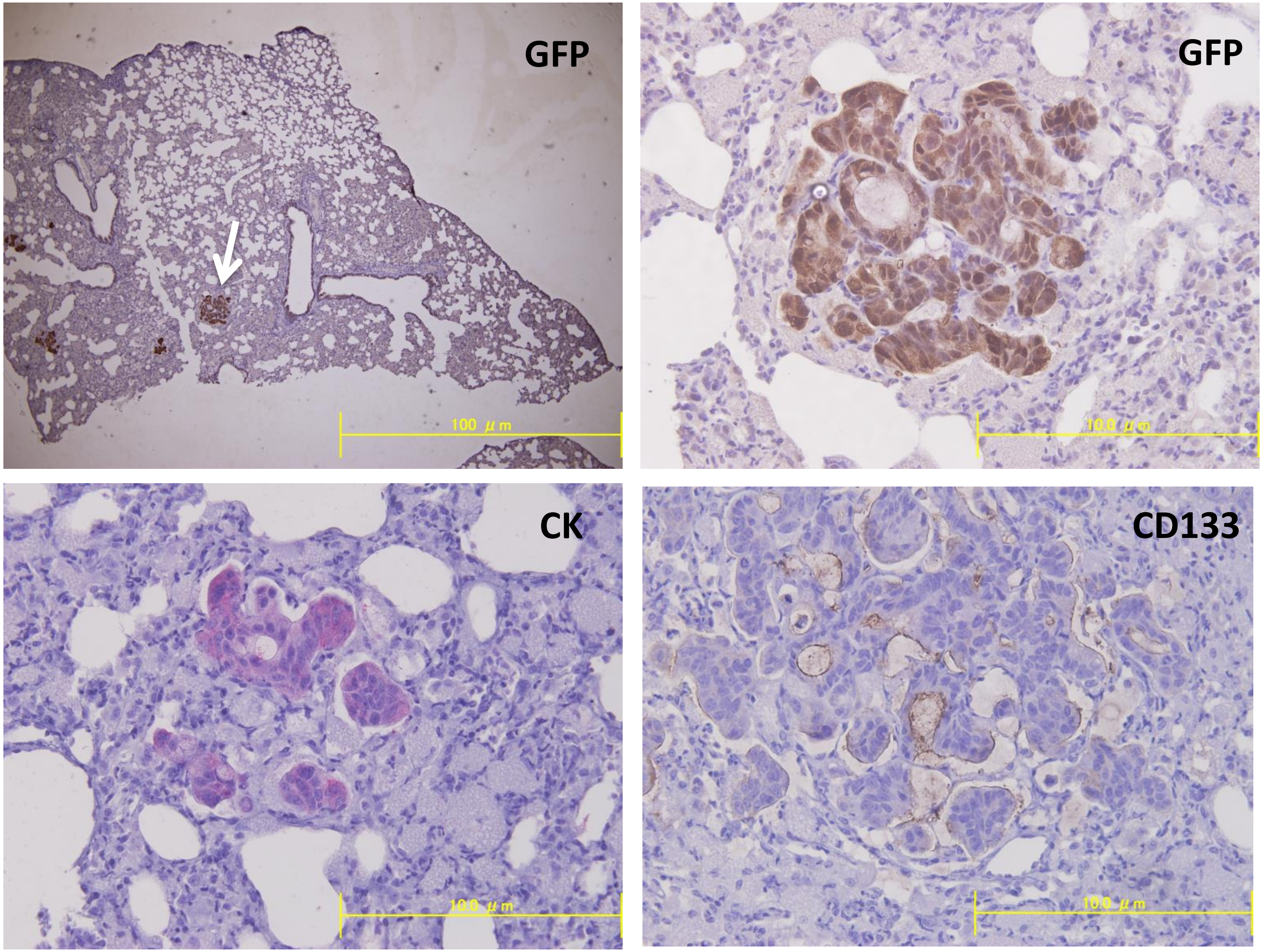

Figure S4: Comparison of tumor-take rates between CD133^{high} and CD133^{KD} cells by subcutaneous xenograft tumor model

Nude mouse with tumors

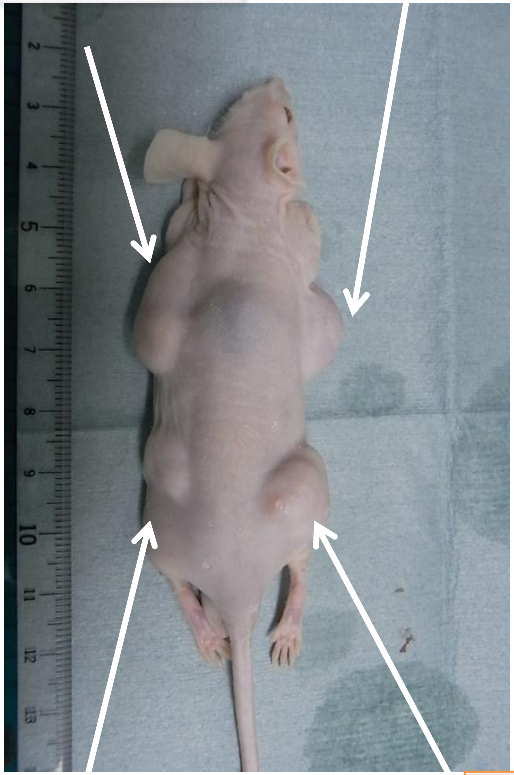

Comparison of tumor-take rate in nude mice between Capan-1M9 and shCD133M9 cells

Number of cells (s.c.)	**Capan-1M9**	**shCD133M9**
100 cells	5/5	5/5
1000 cells	5/5	5/5

(n=5)

Figure S5: Comparison of growth curves among the tumors generated by CD133^{high} and CD133^{KD} cells in xenograft models

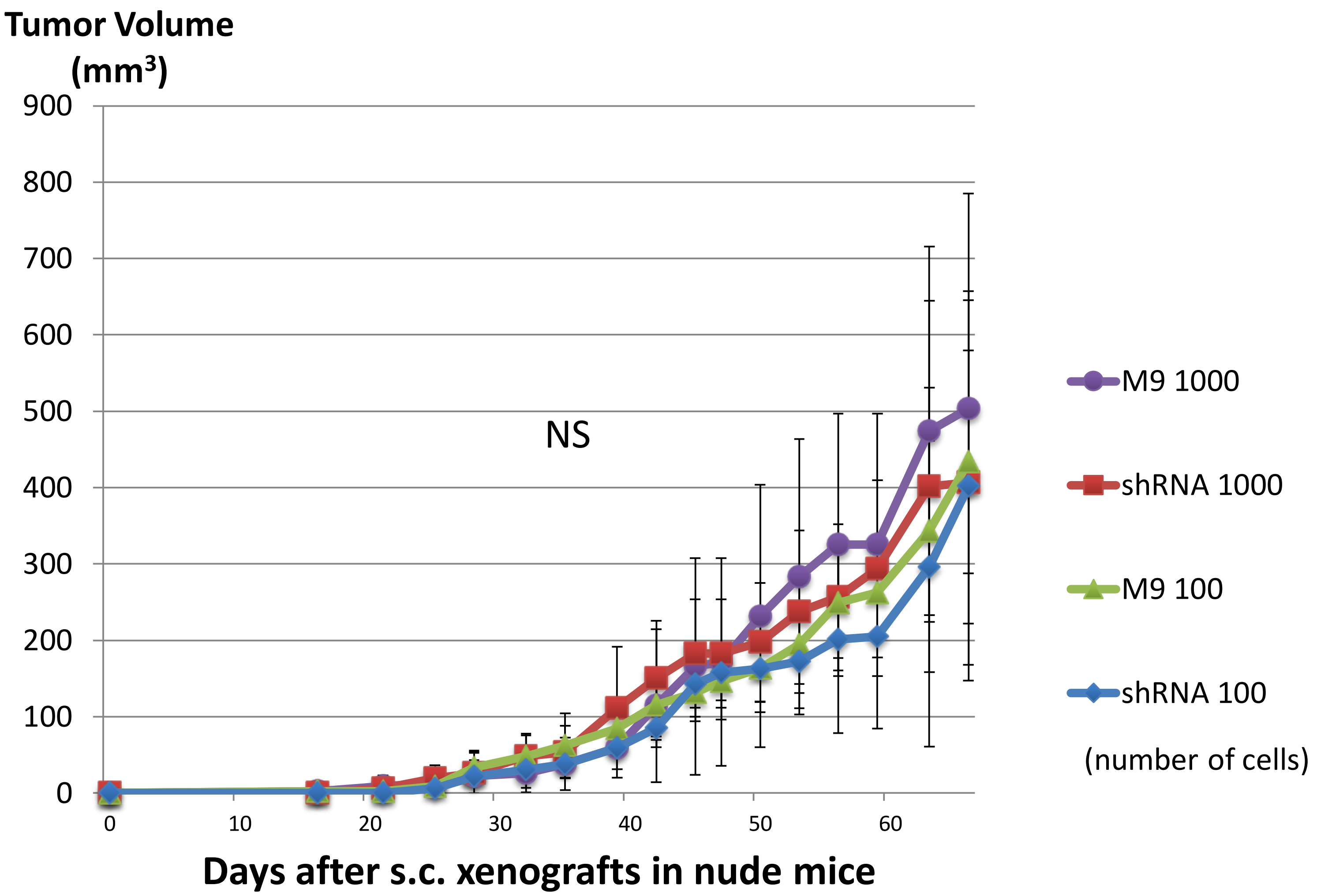

Figure S6: Comparison of four week's tumor volumes between tumors generated by CD133^{high} and CD133^{KD} cells in pancreatic orthotopic model.

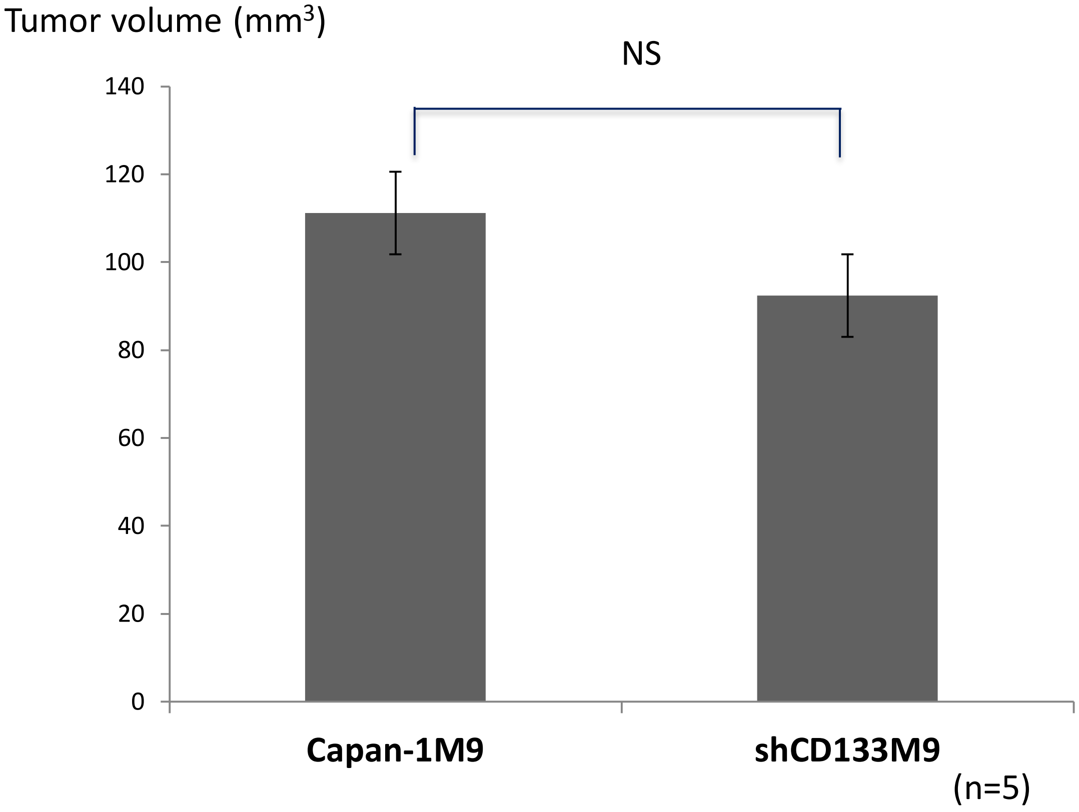

Figure S7: Transduction of shRNACD133 and shRNASlug into Capan1M9 cells. mRNA expression levels of CD133 and Slug were examined after shRNA transduction of CD133 or Slug into Capan1M9 cells (***left***). Fluorescent observation of GFP after shRNA transduction into Capan1M9 cells (***right***).

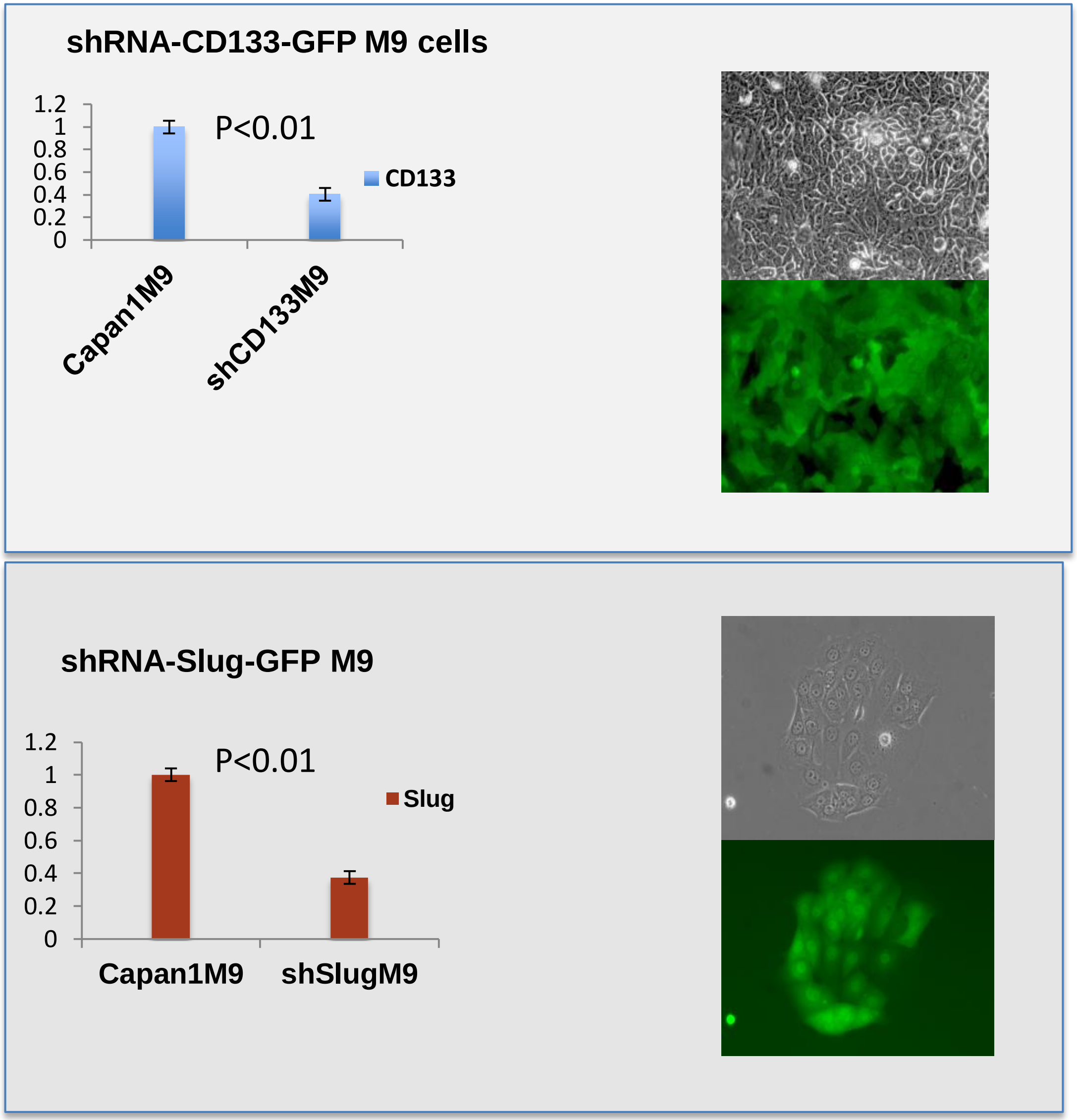

Figure S8: Validation of Slug expression after ERK or TGF inhibitor treatment. Slug mRNA expression was examined after ERK or TGF inhibitor (U0126 or SB431542) treatment.

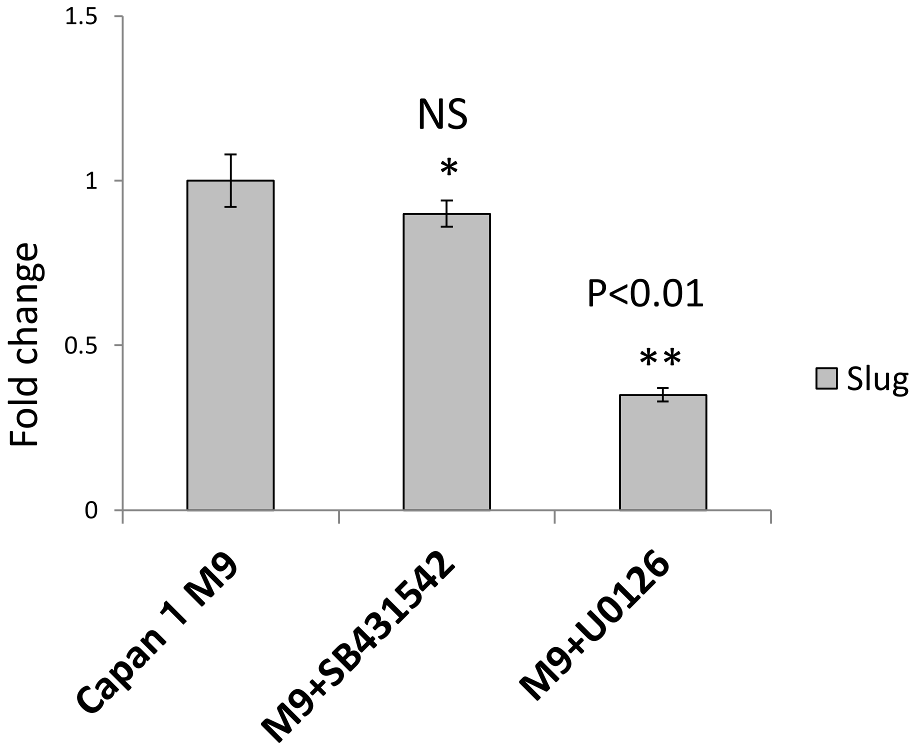

Supplement: Additional file 1: Figure S1 — Examination of CD133 expression before and after shRNA-CD133 transducted into Capan1M9 cells by flow cytometer. Figure S2. In vivo experimental schedules of this study using nude mice. We used Capan1M9-GFP or shCD133M9-GFP cells for these experimental models. We created the lung and liver metastasis models using tail vein or spleen implantation, respectively. In the lung metastasis model, nude mice were sacrificed at every 4 weeks. +: Macroscopically detectable tumor lesion in the organs, -: Undetectable tumor lesion in the organs. Figure S3. The lung metastasis model created by the tail vein injection of Capan1M9-GFP or shCD133M9-GFP cells into nude mice. GFP-positive spots (arrow) indicate micrometastatic lesions in the section of the lung 12 weeks after the tail vein injection of 1 × 10^5^ Capan1M9-GFP cells. Immunohistochemical staining for CK and CD133 was performed to identify micrometastatic lesions. Figure S4. Comparison of tumor-take rates between CD133high and CD133KD cells by subcutaneous xenograft tumor model. Figure S5. Comparison of tumor growth curves in nude mice among the tumors generated by CD133high and CD133KD cells by subcutaneous xenograft tumor model. Figure S6. Comparison of four week’s tumor volumes between tumors generated by CD133high and CD133KD cells in pancreatic orthotopic model. Figure S7. Transduction of shRNACD133 or shRNASlug into Capan1M9 cells. mRNA expression levels of CD133 and Slug were examined after shRNA transduction of CD133 or Slug into Capan1M9 cells (left). Fluorescent observation of GFP after shRNA transduction into Capan1M9 cells (right). Figure S8. Validation of Slug expression after ERK or TGF inhibitor treatment. Slug mRNA expression was examined after ERK or TGF inhibitor (U0126 or SB431542) treatment. [file 1476-4598-13-15-S1.pdf]
