# Supplementary material for: CD133 facilitates epithelial-mesenchymal transition through interaction with the ERK pathway in pancreatic cancer metastasis
Source: Mol Cancer. 2014 Jan 27;13:15. doi: 10.1186/1476-4598-13-15 (PMC3931313; doi:10.1186/1476-4598-13-15)
Supplement: Additional file 2: Table S1 — DNA microarray analysis of epithelial-mesenchymal transition (EMT)-related genes. The data represent a comparison of EMT-related genes expressions between Capan1M9 and shCD133M9 cells by DNA microarray. Table S2. Analysis of EMT-related genes with the following Z scores: Z score ≥ 2 and ratio ≥ 1.5 or Z score ≤ -2 and ratio ≤ 0.66. [file 1476-4598-13-15-S2.pdf]

Table S1

GeneSymbol	Description	Signal		ratio
		M9	shCD133M9	
VIM	Homo sapiens vimentin (VIM), mRNA [NM_003380]	269.405575	14.5888225	0.054151895
SNAI2	Homo sapiens snail homolog 2 (Drosophila) (SNAI2), mRNA [NM_003068]	56.742035	7.067408625	0.124553316
FN1	Homo sapiens fibronectin 1 (FN1), transcript variant 7, mRNA [NM_054034]	23.34250625	3.9644705	0.169839111
IL1RN	Homo sapiens interleukin 1 receptor antagonist (IL1RN), transcript variant 4, mRNA [NM_173843]	15554.5	4020.711	0.258491819
FOXC2	Homo sapiens forkhead box C2 (MFH-1, mesenchyme forkhead 1) (FOXC2), mRNA [NM_005251]	23.70205	6.620899	0.279338665
MITF	Homo sapiens microphthalmia-associated transcription factor (MITF), transcript variant 1, mRNA [NM_198159]	1677.09075	554.6804375	0.330739668
NOTCH1	Homo sapiens notch 1 (NOTCH1), mRNA [NM_017617]	675.14325	247.6606125	0.366826762
CDH2	Homo sapiens cadherin 2, type 1, N-cadherin (neuronal) (CDH2), mRNA [NM_001792]	695.0580281	257.1971135	0.370036894
FZD7	Homo sapiens frizzled family receptor 7 (FZD7), mRNA [NM_003507]	817.834408	320.1853875	0.391503933
ITGAV	Homo sapiens integrin, alpha V (vitronectin receptor, alpha polypeptide, antigen CD51) (ITGAV), transcript variant 1, mRNA [NM_002210]	10146.99361	4141.590421	0.40815936
MMP9	Homo sapiens matrix metallopeptidase 9 (gelatinase B, 92kDa gelatinase, 92kDa type IV collagenase) (MMP9), mRNA [NM_004994]	181.6547384	75.3131224	0.414594869
TGFB2	Homo sapiens transforming growth factor, beta 2 (TGFB2), transcript variant 2, mRNA [NM_003238]	654.2277412	283.6823875	0.433614122
KRT14	Homo sapiens keratin 14 (KRT14), mRNA [NM_000526]	723.035525	327.4258219	0.452848872
STEAP1	Homo sapiens six transmembrane epithelial antigen of the prostate 1 (STEAP1), mRNA [NM_012449]	2937.4265	1382.112792	0.470518255
TMEM132A	Homo sapiens transmembrane protein 132A (TMEM132A), transcript variant 1, mRNA [NM_017870]	11660.30125	6088.394125	0.522147241
KRT7	Homo sapiens keratin 7 (KRT7), mRNA [NM_005556]	205747.9875	108527.3875	0.527477274
PTK2	Homo sapiens PTK2 protein tyrosine kinase 2 (PTK2), transcript variant 1, mRNA [NM_153831]	848.3526125	502.7078875	0.592569505
TSPAN13	Homo sapiens tetraspanin 13 (TSPAN13), mRNA [NM_014399]	2238.7915	1407.64225	0.628750935
ZEB1	Homo sapiens zinc finger E-box binding homeobox 1 (ZEB1), transcript variant 1, mRNA [NM_001128128]	26.05846125	16.5438525	0.634874498
GNG11	Homo sapiens guanine nucleotide binding protein (G protein), gamma 11 (GNG11), mRNA [NM_004126]	202.7561704	139.2596253	0.686832983
ITGB1	Homo sapiens integrin, beta 1 (fibronectin receptor, beta polypeptide, antigen CD29 includes MDF2, MSK12) (ITGB1), transcript variant 1E, mRNA [NM_133376]	1941.083113	1362.83509	0.702100328
CAMK2N1	Homo sapiens calcium/calmodulin-dependent protein kinase II inhibitor 1 (CAMK2N1), mRNA [NM_018584]	5600.74683	4019.404653	0.717655123
EGFR	Homo sapiens epidermal growth factor receptor (EGFR), transcript variant 1, mRNA [NM_005228]	289.3324763	219.9080181	0.760053005
COL5A2	Homo sapiens collagen, type V, alpha 2 (COL5A2), mRNA [NM_000393]	115.58975	87.9014825	0.760460876
PLEK2	Homo sapiens pleckstrin 2 (PLEK2), mRNA [NM_016445]	5031.790125	3903.478625	0.775763402
ITGA5	Homo sapiens integrin, alpha 5 (fibronectin receptor, alpha polypeptide) (ITGA5), mRNA [NM_002205]	174.6968625	136.31525	0.780295926
ILK	Homo sapiens integrin-linked kinase (ILK), transcript variant 3, mRNA [NM_001014795]	2126.26939	1718.618748	0.80827893
SNAI1	Homo sapiens snail homolog 1 (Drosophila) (SNAI1), mRNA [NM_005985]	128.0035525	104.0177297	0.81261596
MSN	Homo sapiens moesin (MSN), mRNA [NM_002444]	536.432725	479.7053125	0.894250649
F11R	Homo sapiens F11 receptor (F11R), mRNA [NM_016946]	28024.39125	26077.105	0.930514592
CAV2	Homo sapiens caveolin 2 (CAV2), transcript variant 1, mRNA [NM_001233]	607.0546613	605.8412483	0.998001147
CDH1	Homo sapiens cadherin 1, type 1, E-cadherin (epithelial) (CDH1), mRNA [NM_004360]	9811.239324	10611.02271	1.08151706
TGFB1	Homo sapiens transforming growth factor, beta 1 (TGFB1), mRNA [NM_000660]	820.4119617	897.561245	1.094037248
SERPINE1	Homo sapiens serpin peptidase inhibitor, clade E (nexin, plasminogen activator inhibitor type 1), member 1 (SERPINE1), transcript variant 1, mRNA [NM_000602]	13.14648905	15.30358359	1.164081416
TIMP1	Homo sapiens TIMP metallopeptidase inhibitor 1 (TIMP1), mRNA [NM_003254]	3068.434625	3628.826375	1.182631152
SNAI3	Homo sapiens snail homolog 3 (Drosophila) (SNAI3), mRNA [NM_178310]	86.723435	104.0595921	1.199901643
ESR1	Homo sapiens estrogen receptor 1 (ESR1), transcript variant 1, mRNA [NM_000125]	28.5033525	40.367195	1.416226214
OCLN	Homo sapiens occludin (OCLN), transcript variant 1, mRNA [NM_002538]	432.1801625	643.668125	1.489351388
IGFBP4	Homo sapiens insulin-like growth factor binding protein 4 (IGFBP4), mRNA [NM_001552]	446.6832875	706.3216625	1.581258315
FGFBP1	Homo sapiens fibroblast growth factor binding protein 1 (FGFBP1), mRNA [NM_005130]	2031.222625	3695.347	1.819272272
ERBB3	Homo sapiens v-erb-b2 erythroblastic leukemia viral oncogene homolog 3 (avian) (ERBB3), transcript variant 1, mRNA [NM_001982]	3384.575438	6670.921	1.970977194
DSP	Homo sapiens desmoplakin (DSP), transcript variant 1, mRNA [NM_004415]	3972.784375	9276.67825	2.335057072
JAG1	Homo sapiens jagged 1 (JAG1), mRNA [NM_000214]	76.85549875	206.2662629	2.683819197
CALD1	Homo sapiens caldesmon 1 (CALD1), transcript variant 1, mRNA [NM_033138]	146.2501125	527.5935875	3.607474746
DSC2	Homo sapiens desmocollin 2 (DSC2), transcript variant Dsc2a, mRNA [NM_024422]	147.7242	724.674875	4.905593498
RGS2	Homo sapiens regulator of G-protein signaling 2, 24kDa (RGS2), mRNA [NM_002923]	308.1948151	1625.207484	5.273312218
BMP7	Homo sapiens bone morphogenetic protein 7 (BMP7), mRNA [NM_001719]	4.928789875	26.6732825	5.411730501
WNT11	Homo sapiens wingless-type MMTV integration site family, member 11 (WNT11), mRNA [NM_004626]	71.56063	396.12025	5.53544945
TCF4	Homo sapiens transcription factor 4 (TCF4), transcript variant 2, mRNA [NM_003199]	34.24982982	192.5447125	5.621771364
PDGFRB	Homo sapiens platelet-derived growth factor receptor, beta polypeptide (PDGFRB), mRNA [NM_002609]	3.724214625	35.3407475	9.489449739

Table S2

GeneSymbol	Description	Signal		ratio	Zscore
		M9	shCD133M9		
VIM	Homo sapiens vimentin (VIM), mRNA [NM_003380]	269.405575	14.5888225	0.054151895	-3.250428181
SNAI2	Homo sapiens snail homolog 2 (Drosophila) (SNAI2), mRNA [NM_003068]	56.742035	7.067408625	0.124553316	-2.024294618
IL1RN	Homo sapiens interleukin 1 receptor antagonist (IL1RN), transcript variant 4, mRNA [NM_173843]	15554.5	4020.711	0.258491819	-3.872556323
MITF	Homo sapiens microphthalmia-associated transcription factor (MITF), transcript variant 1, mRNA [NM_198159]	1677.09075	554.6804375	0.330739668	-2.429942151
NOTCH1	Homo sapiens notch 1 (NOTCH1), mRNA [NM_017617]	675.14325	247.6606125	0.366826762	-2.201136756
CDH2	Homo sapiens cadherin 2, type 1, N-cadherin (neuronal) (CDH2), mRNA [NM_001792]	695.0580281	257.1971135	0.370036894	-2.181885883
FZD7	Homo sapiens frizzled family receptor 7 (FZD7), mRNA [NM_003507]	817.834408	320.1853875	0.391503933	-2.057289317
ITGAV	Homo sapiens integrin, alpha V (vitronectin receptor, alpha polypeptide, antigen CD51) (ITGAV), transcript variant 1, mRNA [NM_002210]	10146.99361	4141.590421	0.40815936	-2.098302212
KRT7	Homo sapiens keratin 7 (KRT7), mRNA [NM_005556]	205747.9875	108527.3875	0.527477274	-3.221719312
DSC2	Homo sapiens desmocollin 2 (DSC2), transcript variant Dsc2a, mRNA [NM_024422]	147.7242	724.674875	4.905593498	2.406117503
RGS2	Homo sapiens regulator of G-protein signaling 2, 24kDa (RGS2), mRNA [NM_002923]	308.1948151	1625.207484	5.273312218	3.688176772
WNT11	Homo sapiens wingless-type MMTV integration site family, member 11 (WNT11), mRNA [NM_004626]	71.56063	396.12025	5.53544945	2.589904223
PDGFRB	Homo sapiens platelet-derived growth factor receptor, beta polypeptide (PDGFRB), mRNA [NM_002609]	3.724214625	35.3407475	9.489449739	2.069150579
